# Comparative analysis of cataract surgery outcomes in elderly Jewish and Arab patients from Northern Israel, a retrospective cohort study

**DOI:** 10.3389/fopht.2026.1942047

**Published:** 2026-09-24

**Authors:** Shadi Boqaaiya, Diana Nujedat, Yuval Cohen, Srour Ward, Emad Ghnem, Adi Sharabi Nov, Karine Beiruti Wiegler, Otzem Chassid

**Affiliations:** 1 Department of Ophthalmology, Ziv Medical Center, Safed, Israel; 2 Azrieli Faculty of Medicine, Bar Ilan University, Safed, Israel; 3 Department of Ophthalmology, Galilee Medical Centre, Nahariyah, Israel; 4 Research Wing, Ziv Medical Center, Safed, Israel; 5 Tel-Hai University of Kiryat Shmona and the Galilee, Tel Hai, Israel; 6 Department of Internal Medicine B, Galilee Medical Centre, Nahariyah, Israel

**Keywords:** cataract surgery, elderly patients, ethnic disparities, health equity, visual outcomes

## Abstract

**Purpose:**

To compare postoperative visual recovery and complications after cataract surgery between Jewish and Arab elderly patients in Northern Israel within a shared healthcare system.

**Methods:**

A retrospective study was conducted at Ziv Medical Center, Safed, Israel, involving 251 patients aged 60 years or older who underwent cataract surgery in 2023. All surgeries were performed under standardized departmental protocols, carried out by senior attending surgeons or supervised senior residents. Demographic characteristics, comorbidities, cataract type, postoperative complications and best-corrected visual acuity (BCVA) were extracted from electronic medical records. Visual outcomes were compared at baseline, day 1, and day 30 postoperatively. All statistical analyses were performed using IBM SPSS Statistics version 31, with continuous variables compared via Mann-Whitney U test and categorical variables via chi-square or Fisher’s exact test, as appropriate.

**Results:**

Baseline age, sex, and preoperative visual acuity were similar between Jewish (n=153) and Arab (n=98) patients. Of the 153 Jewish patients, 77 (50.3%) were male and 76 (49.7%) were female. Of the 98 Arab patients, 49 (50.0%) were male and 49 (50.0%) were female (p = 0.960). Arab patients had higher rates of diabetes (54.1% vs. 35.9%, p=0.005) and hypertension (70.4% vs. 58.2%, p=0.050). Jewish patients demonstrated significantly better postoperative BCVA at 30 days (median LogMAR 0.5 vs. 0.7, p=0.0032). Complications were more common in the Arab cohort, particularly corneal edema (43.9% vs. 15.7%, p<0.001) and posterior capsule rupture (11.2% vs. 3.3%, p=0.012).

**Conclusion:**

Ethnic disparities were observed in short-term outcomes despite a uniform surgical environment within the same tertiary facility. These differences are more plausibly explained by structural, socioeconomic, and comorbidity-related determinants than by any biological difference associated with ethnicity per se. Further multicenter, prospective studies with longer follow-up are required to clarify the multifactorial causes of outcome variation and guide interventions to promote equity in cataract care.

## 1 Introduction

Cataract, defined as partial or complete clouding of the eye’s normally transparent lens or its capsule, is the leading cause of reversible visual impairment in the elderly worldwide (1, 2). While initially slow to progress, cataracts tend to worsen with age, particularly after the fourth or fifth decade, ultimately interfering with daily functioning (3). Nearly half of individuals in their early 70s and almost all in their 90s develop clinically significant cataracts. The National Eye Institute estimates that 38 million people will be affected by 2030 (4), with cataracts accounting for 50% of blindness cases in low- and middle-income countries compared to only 5% in high-income regions (5).

Cataract surgery remains the most effective treatment, offering significant improvements in vision and quality of life. However, as global life expectancy increases, so does surgical demand and the need to understand age-related risks (6). Complications, though uncommon with modern techniques, include posterior capsule rupture, corneal edema, and retinal detachment, significantly impacting outcomes and patient quality of life (2, 7–11).

Ethnicity has been shown to influence cataract prevalence, severity, and surgical outcomes, with disparities in access to care and postoperative results reported across ethnic groups (12). However, limited data exists on outcomes within multiethnic populations treated within the same healthcare system. Age is a related but separate axis of disparity: a retrospective study at Soroka University Medical Center, Israel, revealed more advanced cataracts and worse visual outcomes among late elderly patients compared to middle elderly patients (6), underscoring that patient-level factors beyond surgical technique can shape outcomes even within a single system. Whether comparable disparities exist along ethnic lines, independent of, or interacting with, age, remains poorly characterized.

To date, no study has directly compared cataract surgical outcomes between Jewish and Arab patients treated within a single, shared tertiary institution in Northern Israel, a region marked by close geographic proximity but documented disparities in socioeconomic status, health literacy, and chronic disease burden between these populations (13). This gap limits understanding of whether equal access to a shared surgical facility is sufficient to produce equitable outcomes, or whether upstream determinants continue to drive disparity even under uniform institutional care.

Clarifying the drivers of these disparities carries direct clinical and policy significance: cataract surgery is among the most commonly performed procedures worldwide, and even modest differences in complication rates or visual recovery, translate into a substantial cumulative burden of preventable visual morbidity at the population level. Identifying whether such disparities are modifiable, through targeted comorbidity management, patient education, or adherence support, is therefore essential to informing equitable, evidence-based interventions in ophthalmic care.

This study examines demographic characteristics, clinical features, and postoperative outcomes of cataract surgery between Jewish and Arab populations in Northern Israel, aiming to elucidate ethnicity-specific disparities and advance understanding of factors influencing cataract surgical outcomes among diverse populations.

## 2 Materials and methods

### 2.1 Subjects and measurements

This single-center, retrospective cohort study included patients who underwent cataract surgery between January 1st, 2023, and December 31st, 2023, in the Department of Ophthalmology at Ziv Medical Center, Safed, Israel. Patient data were collected from the hospital’s electronic medical records (EMR) database, including demographic variables (age, sex and ethnicity), systemic and ocular comorbidities, cataract type classification (nuclear sclerotic (NS), cortical, and posterior sub-capsular cataracts), best-corrected visual acuity (BCVA), intraoperative complications, and postoperative outcomes. Inclusion criteria required patients to have no history of prior ocular surgery, and a pre-operative best-corrected visual acuity (BCVA) of 6/12 or worse. Exclusion criteria included age less than 60 years, evidence of corneal diseases (e.g., corneal dystrophies, ectasias), glaucoma, or retinal pathologies (e.g., Diabetic Retinopathy, Hypertensive Retinopathy, Retinal Detachment (RD), or Age-related macular degeneration (AMD)), and patients with less than one month of postoperative follow-up. All included eyes had a clear cornea, with no clinical evidence of corneal guttae, corneal edema, known corneal dystrophy, or corneal ectasia.

All cataract surgeries were performed using standard phacoemulsification with the Centurion Vision System (Alcon Laboratories, Fort Worth, TX, USA). Surgical procedures followed the department’s standardized protocol and were performed by senior attending surgeons or supervised senior residents according to department’s standardized protocol. All visual acuity values reported in the study represent BCVA. Corneal edema was diagnosed by slit-lamp examination at the postoperative visit and recorded according to the operating ophthalmologist’s clinical assessment.

The study included all eligible consecutive patients who underwent cataract surgery during the study period and had complete medical records (**Table 1**). Patients were categorized into two ethnic groups, Jewish and Arab. This stratification enabled a more detailed comparative analysis of surgical outcomes across different demographic groups.

**Table 1:** Demographic characteristics of patients undergoing cataract surgery by ethnicity.

Characteristics	Jews (n=153)	Arabs (n= 98)	p value
Age, Me (IQR)	73 (68-79)	73 (69-80)	0.829
Gender, Male, n (%)	77 (50.3)	49 (50.0)	0.960
Comorbidities and risk factors			
Hypertension, n (%)	89 (58.2)	69 (70.4)	**0.050**
Diabetes mellitus, n (%)	55 (35.9)	53 (54.1)	**0.005**
COPD, n (%)	16 (10.5)	10 (10.2)	0.949
Dyslipidemia, n (%)	43 (28.1)	37 (37.8)	0.109

Me, median; IQR, interquartile range; COPD, chronic obstructive pulmonary disease.

Bold values indicate statistical difference (P<0.05).

### 2.2 Statistical analysis

Statistical analyses were conducted using IBM SPSS Statistics version 31 (IBM Corp., Armonk, NY, USA). Continuous variables were presented as medians (Me) and interquartile ranges (IQR), since the data were not normally distributed according to the Kolmogorov-Smirnov test. Categorical variables were summarized as frequencies and percentages. Differences in continuous variables between groups were assessed using the Mann–Whitney non-parametric test. Categorical variables were compared using Pearson’s chi-square (χ²) test or Fisher’s exact test, as appropriate. All statistical tests were two-sided, and a p-value of <0.05 was considered statistically significant.

## 3 Results

The study included 251 patients who underwent cataract surgery in 2023, comprising 153 (61%) of Jewish ethnicity and 98 (39%) of Arab ethnicity. The median age was 73 years for both ethnicities. Gender distribution was nearly identical between the groups: 50.3% male in the Jewish cohort, compared to 50.0% male in the Arab cohort (p = 0.960). These results indicate that potential differences in postoperative outcomes are unlikely to be influenced by confounding differences in age or gender between the groups.

A comparison of systemic comorbidities between the Jewish and Arab populations revealed significant differences in the prevalence of certain conditions. Hypertension was more common in the Arab cohort (p = 0.050), as was diabetes mellitus (p = 0.005). In contrast, no significant differences were observed for chronic obstructive pulmonary disease (COPD) (p = 0.949) or dyslipidemia (p = 0.109).

Visual acuity measurements were assessed at two time points: preoperatively and on postoperative day 30, to evaluate and compare visual outcomes between the Jewish and Arab patient groups (**Table 2**). Preoperative visual acuity was statistically comparable between groups, with both exhibiting a median LogMAR of 0.7 (p = 0.697), indicating no significant baseline differences in ocular function. However, by postoperative day 30, this difference became statistically significant: The Jewish group demonstrated a lower mean LogMAR of 0.5 versus 0.7 in the Arab group (p = 0.032), reflecting a superior visual improvement over time. These findings suggest that while both groups began with equivalent visual acuity, the Jewish population experienced a greater improvement by the 30-day follow-up. The results highlight a potential disparity in long-term visual recovery, warranting further investigation into postoperative care adherence, access to follow-up, and broader systemic factors that may influence surgical outcomes across different population groups.

**Table 2:** Visual acuity measurements, cataract types, and postoperative complications according to ethnicity group.

Clinical variables	Jews (n=153)	Arabs (n= 98)	p value
Visual Acuity			
Snellen, Me (IQR)			
Before surgery	36 (20-60)	36 (18-60)	0.724
30 days from surgery 30	20 (12-45)	30 (15-60)	**0.016**
LogMar, Me (IQR)			
Before surgery	0.7 (0.5-1.0)	0.7 (0.4-1.0)	0.697
30 days from surgery 30	0.5 (0.3-0.8)	0.7 (0.4-1.0)	**0.032**
Cataract type, n (%)			
PSC	34 (22.2)	30 (30.6)	0.137
Cortical	53 (34.6)	43 (43.9)	0.142
NS	150 (98.0)	93 (94.9)	0.167
Complications, n (%)			
None	101 (66)	28 (28.6)	**<0.001**
Retained lens fragments	8 (5.2)	3 (3.3)	0.543
Posterior capsular opacification	4 (2.6)	7 (7.1)	0.115
Posterior capsular rupture	5 (3.3)	11 (11.2)	**0.012**
IOL Dislocation	7 (4.6)	3 (3.1)	0.745
Corneal edema	24 (15.7)	43 (43.9)	**<0.001**
Elevated IOP	6 (3.9)	4 (4.1)	0.950

Me, median; IQR, interquartile range; PSC, Posterior sub-capsular; CS, Cortical; NS, Nuclear sclerosis; IOP, intraocular pressure; IOL, intraocular lens.

No differences were observed in the prevalence and severity of specific cataract types between the Jewish and Arab populations, as detailed in **Table 2**.

The incidence of postoperative complications following cataract surgery significantly differed between the Jewish and Arab populations (p<0.001), as summarized in **Table 2**. In the Jewish cohort, 66% of patients experienced no postoperative complications, while 34% reported at least one complication. In contrast, only 28.6% of patients in the Arab cohort experienced no complications, suggesting a higher overall complication rate in this group. The most frequently observed complication was corneal edema. It was significantly more prevalent among Arab patients, affecting 43.9% compared to 15.7% in the Jewish cohort (p<0.001). Posterior capsular rupture was also more common among Arab patients (11.2% vs. 3.3%, p=0.012). The rates of retained lens fragments (3.3% vs. 5.2%), posterior capsular opacification (7.1% vs. 2.6%), IOL dislocation (3.1% vs. 4.6%) and elevated IOP (4.1% vs. 3.9%) did not differ significantly between the two groups. **Figure 1** illustrates the frequency of postoperative complications following cataract surgery according to ethnicity.

**Figure 1:**
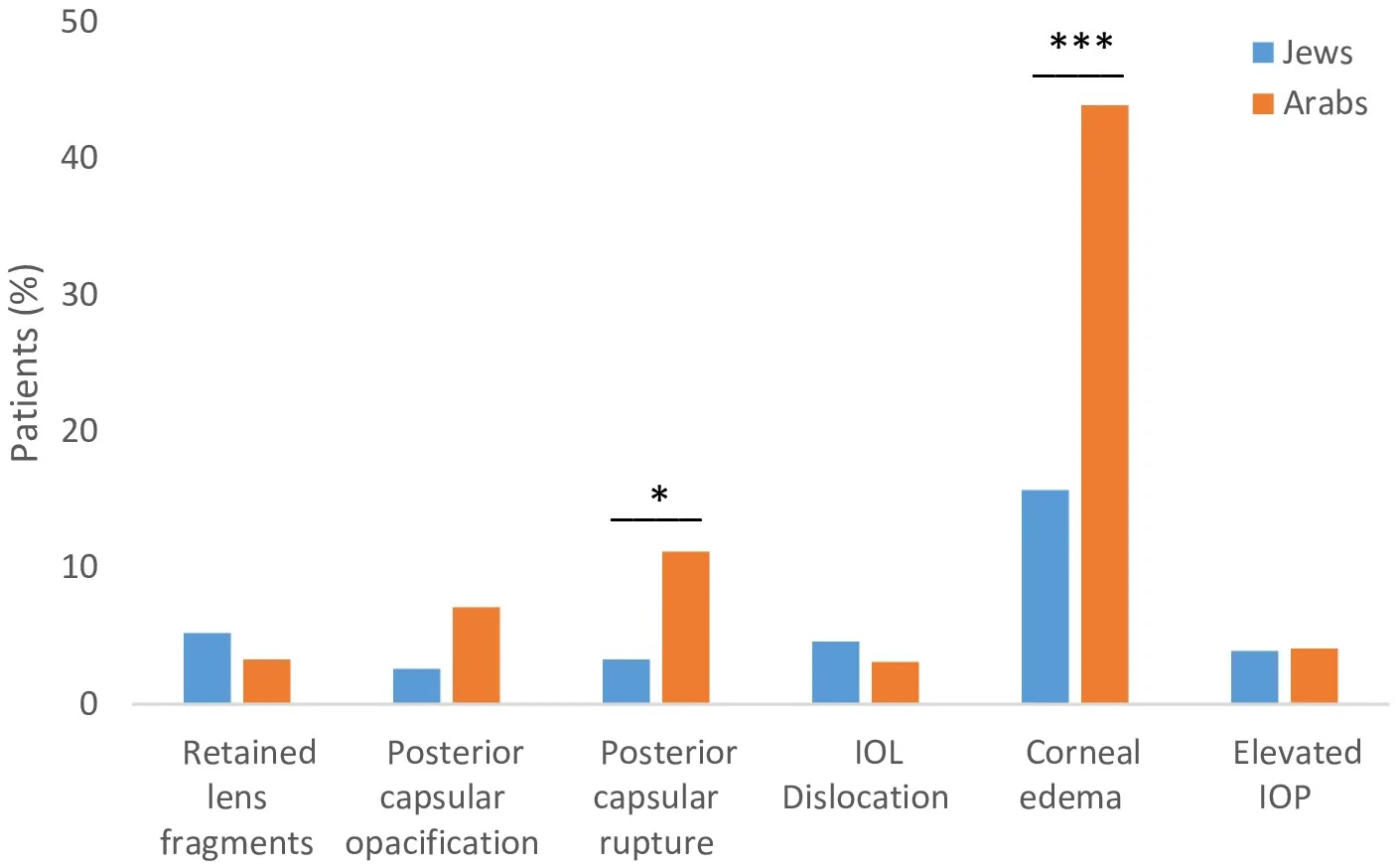
Comparison of postoperative complication rates between Jewish and Arab patients following cataract surgery. * Statistically significant, *** Highly significant.

Multivariable logistic regression was performed to assess the association between postoperative complications and ethnicity, adjusting for age, gender, hypertension, and diabetes (**Table 3**). Arab ethnicity was independently associated with a higher likelihood of postoperative complications (adjusted OR 4.74, 95% CI 2.68–8.39). Male patients suffered 1.72 times more from postoperative complications than female patients (95% CI: 1.00–2.97, p=0.050). Diabetic patients suffered 1.71 times more from postoperative complications compared to non-diabetic patients, although this association was borderline-significant (95% CI: 0.97–3.01, p=0.066). Age and hypertension were not found to be significantly associated with postoperative complications.

**Table 3:** Multivariable logistic regression to assess the association between any postoperative complication and ethnicity, adjusting for age, gender, HTN, and diabetes.

Variables	OR	95% CI	p value
Ethnicity	4.74	2.68-8.39	**<0.001**
Gender	1.72	1.00-2.97	**0.050**
Age	1.00	0.96-1.04	0.841
Hypertension	0.75	0.42-1.34	0.327
Diabetes mellitus	1.71	0.97-3.01	**0.066**

OR, odds ratio; CI, confidence interval.

Bold values indicate statistical difference (P<0.05).

## 4 Discussion

This study identified significant differences in cataract surgery outcomes between Jewish and Arab elderly patients in Northern Israel despite treatment within the same healthcare system. The findings align with global literature on ethnic and socioeconomic influences on visual outcomes but provide novel, localized evidence from Northern Israel, a unique multicultural setting where both groups access care under the same institutional protocols.

Importantly, multivariable logistic regression demonstrated that Arab ethnicity remained independently associated with postoperative complications after adjustment for age, sex, hypertension, and diabetes. This finding suggests that the observed disparity cannot be explained solely by differences in these measured baseline characteristics, although residual confounding from unmeasured factors cannot be excluded. Our finding that Arab ethnicity was independently associated with postoperative complications parallels broader reports of ethnic disparities in ophthalmic surgical outcomes (12), though direct comparators for cataract surgery specifically, within a single shared institution, are scarce. This contrasts with studies from more resourced, homogeneous healthcare systems where ethnicity-based outcome gaps are less pronounced once comorbidities are controlled for, suggesting that the disparity observed here may reflect factors specific to the socioeconomic and healthcare-access landscape of Northern Israel rather than a universal pattern.

We wish to emphasize that ethnicity in this context is best understood as a social and structural variable, an indicator for differences in comorbidity burden, socioeconomic status, health literacy, and healthcare access and utilization patterns, rather than as an indicator of any biological or genetic difference between groups. This framing is consistent with a substantial body of literature on ethnic health disparities in Israel, which attributes such gaps primarily to structural and socioeconomic determinants rather than innate biological differences (13–16).

Arab patients had a higher prevalence of diabetes and hypertension, which are recognized risk factors for more complex cataract surgery and less favorable postoperative recovery. The higher prevalence of diabetes in the Arab cohort may partly reflect dietary and lifestyle patterns reported in regional epidemiological studies, together with broader social determinants of health and differences in health awareness (17, 18). However, these factors were not directly assessed in the present study, and no causal inferences can be drawn. Because objective cataract grading and phacoemulsification parameters were unavailable, the contribution of cataract severity cannot be determined from the present study.

Finally, our reliance on 30-day outcomes limits the ability to capture longer-term visual recovery and complication trajectories. Corneal edema, the most common early complication, may resolve over weeks to months, potentially narrowing the disparity observed at the short-term endpoint. Future studies should include a 3-12-month follow-up to assess final visual acuity, posterior capsule opacification progression, and quality-of-life outcomes.

Socioeconomic factors may have contributed to the disparities observed in this study. As recognized determinants of healthcare access and quality (19), socioeconomic differences may influence the timing of presentation, access to ophthalmic care, and postoperative recovery. However, because objective cataract grading was unavailable, the impact of cataract severity cannot be determined in the present study.

Postoperative visual outcomes further underscore these differences. While both cohorts had comparable baseline visual acuity, the Jewish group exhibited significantly greater visual improvement by postoperative day 30. The Arab cohort experienced a higher rate of mild yet vision-affecting complications, particularly corneal edema and posterior capsular rupture.

Another factor that may underlie postoperative disparities is adherence to prescribed topical regimens. Literature suggests that poor compliance with anti-inflammatory and antibiotic eye drops is associated with higher complication rates, including persistent corneal edema and cystoid macular edema (20). While our study did not directly assess adherence, this remains a plausible hypothesis warranting further prospective evaluation. While Nouraeinejad (20) established a general link between poor postoperative eye drop adherence and complications such as corneal edema, ethnicity-stratified adherence data are lacking, both in the Israeli context and more broadly. This represents an important area for future prospective study using objective adherence measures (e.g., medication possession ratios or electronic drop monitors).

Despite both populations receiving care within the same institutional setting, unmeasured patient-level factors may have contributed to the observed disparities. Differences in health literacy, language proficiency, understanding of postoperative instructions, and adherence to topical medications may influence visual recovery and complication rates (21). In addition, cultural factors influencing healthcare utilization and follow-up attendance may also contribute to postoperative outcomes (22). Although these variables were not assessed in the present study, they represent important areas for future investigation.

The timing of surgical intervention is an important determinant of cataract surgery outcomes. Although the present study did not assess referral patterns or time to surgery, delayed presentation and barriers to accessing ophthalmic care represent plausible explanations for the observed disparities and warrant investigation in future prospective studies.

While this study focused primarily on clinical outcomes, a more comprehensive evaluation including long-term visual function and patient-reported quality-of-life metrics is essential to fully assess the impact of the observed disparities. Future research should adopt prospective designs and include larger, more diverse cohorts to further elucidate the multifactorial nature of cataract outcomes. Additionally, examining the influence of socioeconomic factors, cultural beliefs, eye drop adherence, and overall postoperative compliance may provide valuable insights for optimizing care in underserved populations.

Overall, our findings suggest that disparities in cataract outcomes are multifactorial and likely reflect differences in systemic comorbidities, socioeconomic factors, health literacy, postoperative adherence, and other unmeasured determinants rather than ethnicity per se.

This study has several limitations. First, its retrospective, single-center design restricts generalizability and introduces potential selection bias. Second, follow-up was limited to 30 days, which may underestimate long-term recovery and complication rates, particularly for transient conditions such as corneal edema. Third, ocular comorbidities such as amblyopia, subtle retinal disease, or endothelial dystrophies were not comprehensively captured in our dataset and may have differed between groups. Fourth, socioeconomic status, health literacy, and adherence to postoperative eye drops were not directly evaluated; thus, ethnicity may serve only as a proxy for broader structural determinants of health. Fifth, this study did not include standardized preoperative lens grading using the Lens Opacities Classification System III (LOCS III). Without objective assessment of nuclear density or cataract hardness, differences in baseline cataract severity between the two ethnic groups cannot be excluded as a contributor to the higher rates of posterior capsule rupture and corneal edema observed in the Arab cohort, as denser or more advanced cataracts are independently associated with longer phacoemulsification time, greater ultrasound energy delivery, and increased intraoperative complication risk. Additionally, although all surgeries followed a standardized departmental protocol, inter-surgeon variation in technique, experience level, and operative time was not captured in this dataset and may have contributed to differences in complication rates. Finally, as with all retrospective studies, unmeasured confounders may contribute to the observed differences. Future multicenter, prospective studies with longer follow-up, detailed surgeon-level data, and direct measures of adherence and socioeconomic status are needed to better delineate the drivers of outcome disparities.

This study demonstrates significant disparities in cataract surgery outcomes between Jewish and Arab elderly patients in Northern Israel, despite comparable access to care within the same tertiary hospital and standardized surgical protocols. While both groups began with similar demographic profiles and baseline vision, the Arab cohort exhibited a higher burden of comorbidities such as diabetes and hypertension, and greater rates of short-term complications including corneal edema and posterior capsular rupture. Systemic health factors together with socioeconomic determinants, and postoperative adherence are likely to contribute substantially.

These findings highlight the need for targeted interventions, including earlier cataract detection, optimized management of systemic comorbidities, improved patient education, and strategies to support adherence to postoperative care to ensure equitable surgical outcomes across populations. Future prospective, multicenter studies with longer follow-up and surgeon-level data are essential to clarify the multifactorial causes of disparity and to inform policies that promote health equity in ophthalmology.

## Data Availability

The raw data supporting the conclusions of this article will be made available by the authors, without undue reservation.
